# Aspirated Soybean Mimicking Endobronchial Tumour Removed With a Cryoprobe

**DOI:** 10.1002/rcr2.70366

**Published:** 2025-10-01

**Authors:** Shoichiro Matsumoto, Yuki Takigawa, Ken Sato, Kodai Honda, Mariko Otsuki, Satoko Ido, Mayu Goda, Keisuke Shiraha, Takeru Ichikawa, Suzuka Matsuoka, Jun Nishimura, Hiromi Watanabe, Kenichiro Kudo, Keiichi Fujiwara, Takuo Shibayama

**Affiliations:** ^1^ Department of Respiratory Medicine NHO Okayama Medical Center Okayama Japan

**Keywords:** accidental aspiration, airway foreign bodies, cryotherapy, rigid bronchoscopy, soybean

## Abstract

Adult airway foreign bodies are misdiagnosed as endobronchial tumours on imaging. A 77‐year‐old man with asbestosis presented with a worsening cough. Computed tomography (CT) showed a 12‐mm low‐attenuation right upper‐lobe bronchial lesion with distal bronchiectasis and infiltrates. Magnetic resonance imaging (MRI) suggested a fat‐containing tumour (lipoma or hamartoma). Flexible bronchoscopy revealed a yellowish polypoid mass; poor sedation tolerance necessitated rigid bronchoscopy under general anaesthesia. Using a 1.7‐mm cryoprobe, the lesion was cryoactivated for 10 s and removed en bloc. It proved to be a foreign body resembling a legume and was later confirmed to be an aspirated soybean from 1 month earlier. The fat and water content of soybeans can yield MRI appearances similar to fatty tumours. Distinguishing tumours from foreign bodies by imaging or bronchoscopy alone is challenging. Cryoprobe extraction is effective for hydrated foreign bodies, and aspiration should be considered in the differential diagnosis of endobronchial tumours.

## Graphical abstract

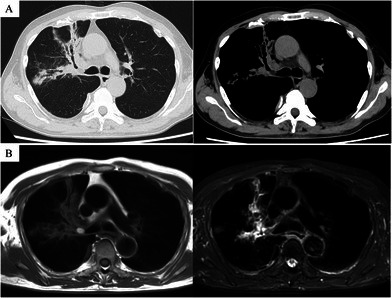

## 1 Introduction

Airway foreign bodies are more commonly found in the right lower lobe, and only approximately 5% are impacted in the right upper lobe [1]. This makes it difficult to strongly suspect them preoperatively. Possible reasons for upper‐lobe impaction include aspiration episodes while supine, bronchomalacia, and excessive airway collapse during coughing [2]. We present a case of an airway foreign body removed by cryoprobe that was initially considered to be endotracheal lipoma or endobronchial hamartoma based on imaging findings.

## 2 Case Report

**FIGURE 1:**
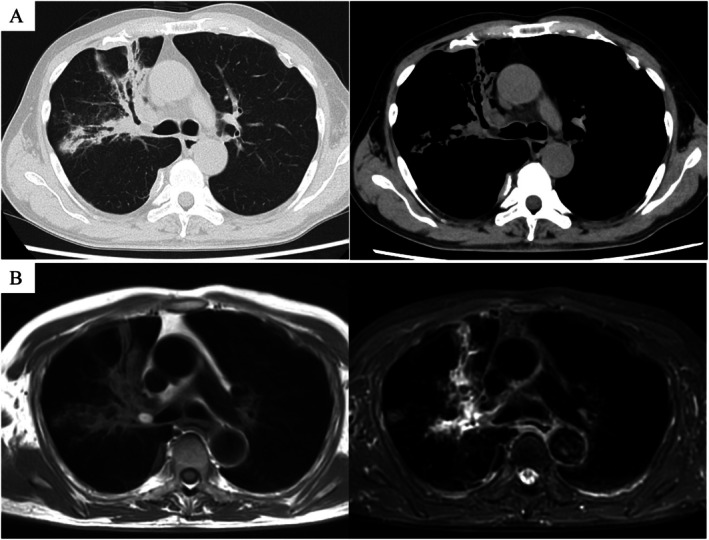
Imaging obtained before bronchoscopy. (A) The CT demonstrated a 12‐mm endobronchial tumour with low attenuation in the right upper lobe bronchus. Bronchiectasis and some infiltrative shadows, suggestive of obstructive pneumonia, were observed. (B) The tumour in the right upper lobe bronchus appeared hyperintense on T1‐weighted magnetic resonance imaging (MRI) and underwent signal suppression on fat‐suppressed T1‐weighted images. The lesion appeared hypointense on STIR images. The radiologist interpreted these findings as suggestive of a fat‐containing tumour.

A 77‐year‐old man presented with a chief complaint of worsening cough. His medical history included asbestosis, postoperative adhesive small bowel obstruction, and postoperative acute appendicitis. He had never smoked. He had a known history of asbestosis and chronic cough. However, his cough worsened in late April 2025, prompting a visit to a respiratory specialist at a previous hospital in May 2025. He was subsequently referred to our department for further evaluation because of abnormal chest computed tomography (CT) findings. The CT demonstrated a 12‐mm endobronchial tumour with low attenuation in the right upper‐lobe bronchus. Bronchiectasis and some infiltrative shadows suggestive of obstructive pneumonia were observed distally (Figure 1A). The mass in the right upper‐lobe bronchus appeared hyperintense on T1‐weighted magnetic resonance imaging (MRI) and underwent signal suppression on fat‐suppressed T1‐weighted images. The lesion appeared hypointense on short tau inversion recovery images. The radiologist interpreted these findings as suggestive of a fat‐containing tumour (Figure 1B).

**FIGURE 2:**
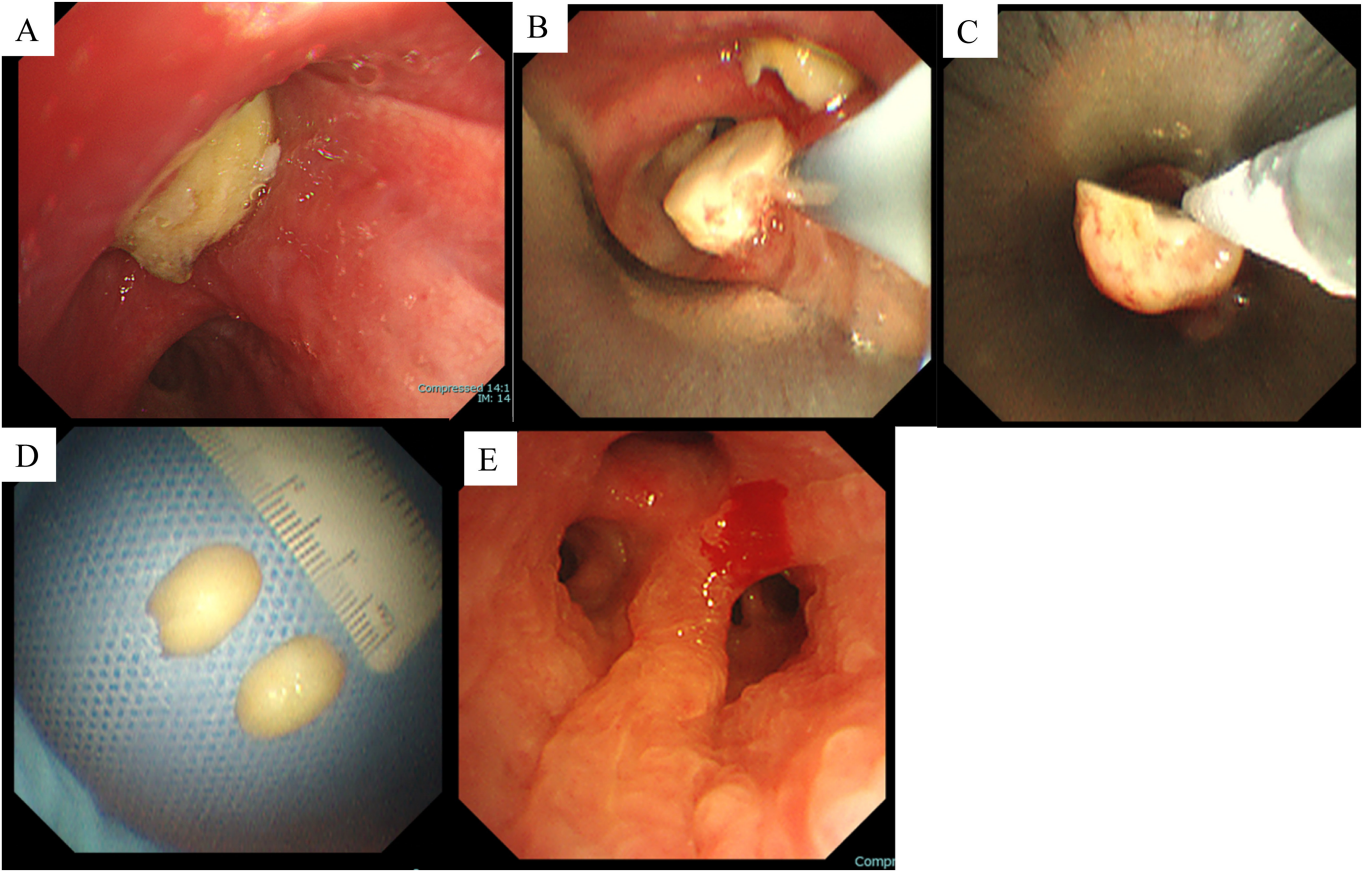
Imaging obtained during bronchoscopy. A yellowish tumour covered with mucus was observed at the proximal right upper lobe bronchus (A). A 1.7‐mm single‐use cryoprobe was applied to the surface of the tumour to freeze it for 10 s. The tumour unexpectedly split in half during this process. Each half was subsequently frozen for 10 s and removed from the airway (B–D). The lesion was found to be a foreign body resembling a legume rather than the initially suspected fat‐containing tumour (D). Residual findings of the foreign body were not observed. The airway was patent, with only mild granulation observed (E).

Diagnostic resection of the lesion at the proximal right upper‐lobe bronchus was deemed necessary. Flexible bronchoscopy was performed under intravenous and topical anaesthesia on day two of hospitalisation. A yellowish tumour covered with mucus was observed at the proximal right upper‐lobe bronchus (Figure 2A). No neovascularization or bleeding tendency was observed during forceps manipulation. The patient responded poorly to sedatives and coughed frequently. Rigid bronchoscopic tumour resection under general anaesthesia was deemed appropriate and scheduled for day four of hospitalisation due to the size of the tumour and poor sedation with intravenous and topical anaesthesia. A rigid bronchoscope (outer diameter, 13.2 mm) and a therapeutic bronchoscope (BF‐1TQ290, Olympus) were used. A 1.7‐mm single‐use cryoprobe was applied to the surface of the tumour to freeze it for 10 s. The tumour unexpectedly split in half during the cryotherapy process. Each half was subsequently frozen for 10 s and removed from the airway (Figure 2B,C). The lesion was found to be a foreign body resembling a legume rather than the initially suspected fat‐containing tumour (Figure 2D). Residual findings of the foreign body were not observed. Only mild granulation with a patent airway was observed (Figure 2E). The total duration for the bronchoscopy was 20 min. Further detailed questioning of the patient revealed that he may have aspirated a large quantity of soybeans approximately 1 month earlier. Therefore, an airway foreign body (aspirated soybean), instead of an endobronchial tumour, was finally diagnosed.

## 3 Discussion

Airway foreign bodies are often identified before bronchoscopy in 30%–50% of cases. Many adult aspiration events remain undiagnosed because patients are unaware of the incident. Even when patients are conscious and oriented, aspiration of small food items may go unrecognised. If a foreign body lodges in the peripheral bronchi, symptoms are often mild and nonspecific—such as cough or chest discomfort—leading to frequent misinterpretation as upper respiratory infection or bronchitis. Habitual alcohol consumption increases aspiration risk even in individuals without a history of cerebrovascular disease, neuromuscular disorders, or aspiration pneumonia; this risk is often unrecognised by patients [3]. Similarly, this patient had no history of significant cognitive decline or neurologic disease, and his interview responses did not suggest an airway foreign body.

At our institution, in addition to performing CT for endobronchial tumours, patients also undergo MRI to help predict intra‐tumoral characteristics and assess submucosal and extramural extension, thereby informing treatment planning. Fat‐suppressed sequences are diagnostic for lipoma, and in carcinoid tumours, combining MRI with CT has been reported to improve diagnostic accuracy [4]. Furthermore, in tracheal leiomyoma, MRI is useful for evaluating the tumour base and determining the appropriateness of surgical resection [5].

The yellowish polypoid tumour covered with mucus observed during bronchoscopy was consistent with previously reported cases of yellowish polyps observed in endobronchial lipomas. The preoperative diagnosis of a bronchial tumour rich in fatty components, such as an endobronchial lipoma or lipomatous hamartoma, was made based on preoperative bronchoscopy, CT, and MRI findings. However, the specimen obtained via cryoprobe was later identified as a soybean. Soybeans generally have a high fat content. The soybean in this case had been boiled, presumably giving it a high‐water content. Mixtures of water and oil produce significant artefacts on MRI [6], therefore, regarding airway foreign bodies, accurate diagnosis may be difficult using imaging modalities including MRI. Cases that required the differentiation of endobronchial tumours from a peanut and screw have been reported [7, 8]. The lesions in both cases were initially presumed to be tumours and only identified as foreign bodies after surgical resection. This suggests the difficulty of definitively distinguishing an endobronchial tumour from an airway foreign body using imaging or preoperative bronchoscopy alone.

Bronchial tumours are typically removed using a high‐frequency snare or cryoprobe at our institution. In our institution's interventional pulmonology practice, peripheral pulmonary lesions are typically frozen for 4–8 s using a 1.7‐mm cryoprobe. For central airway lesions with a high risk of bleeding, freezing is initiated for 3–5 s and then adjusted (3–10 s) based on bleeding status and specimen adequacy [9]. We opted to initially use a cryoprobe because the lesion was comparatively large, and preoperative imaging failed to detect a stalk of the target.

Organic foreign bodies such as peanuts and beans contain moisture, and their surfaces freeze readily, allowing firm adhesion to the cryoprobe. They can therefore often be removed en bloc, reducing the risk of fragmentation or residual material. By contrast, inorganic foreign bodies (e.g., plastic or metal) lack moisture, making adhesion by freezing unlikely; in such cases, forceps or baskets are usually more effective. The soybean was incidentally split into two, and both pieces were completely removed in two passes. Attempting removal with forceps would likely have caused fragmentation, scattering, and retention within the airway; thus, cryoprobe removal was advantageous. The use of a cryoprobe is recommended for effective treatment when differentiating between a tumour and a foreign body, particularly an organic foreign body, is difficult.

In conclusion, a bronchial foreign body (soybean) that was initially considered to be a fat content endobronchial tumour and was safely and completely removed under general anaesthesia using a cryoprobe. This case highlights the need to consider airway foreign bodies when a bronchial tumour is included in the differential diagnosis. Clinicians should obtain detailed clinical history preoperatively and ensure that devices for removing foreign bodies are readily available during bronchoscopic removal.

## Author Contributions

Shoichiro Matsumoto and Yuki Takigawa wrote the manuscript, which was reviewed by all co‐authors. All authors have approved the final version of the manuscript for submission.

## Consent

The authors declare that written informed consent was obtained for the publication of this manuscript and accompanying images and attest that the form used to obtain consent from the patient complies with the journal requirements as outlined in the author guidelines.

## Conflicts of Interest

Yuki Takigawa received lecture fees from AMCO, Japan.

## Data Availability

The data that support the findings of this study are available from the corresponding author upon reasonable request.
